# Assessing the Relationship Between Blubber Thickness and Adipocyte Size in Beluga Whales

**DOI:** 10.3390/ani16040650

**Published:** 2026-02-18

**Authors:** Jordan B. Stewart, Amanda M. Belanger, Cortney A. Watt

**Affiliations:** 1 Arctic Fisheries and Marine Mammal Science Division, Fisheries and Oceans Canada, Winnipeg, MB R3T 2N6, Canada; amanda.belanger2@dfo-mpo.gc.ca (A.M.B.); cortney.watt@dfo-mpo.gc.ca (C.A.W.); 2 Department of Biological Sciences, University of Manitoba, Winnipeg, MB R3T 2N2, Canada; 3 Maurice Lamontagne Institute, Fisheries and Oceans Canada, Mont-Joli, QC G5H 3Z4, Canada; 4 Department of Environment and Geography, University of Manitoba, Winnipeg, MB R3T 2M6, Canada

**Keywords:** *Delphinapterus leucas*, body condition, energy storage, population health

## Abstract

Body condition, the relative amount of energy reserves in an individual, reflects nutritional status and overall health in marine mammals and can indicate the influence of stressors on individuals. Energy in marine mammals is primarily stored as lipids within adipocytes in blubber tissue, making blubber thickness a common proxy for body condition. However, blubber also serves structural roles, complicating its use as a body condition indicator. Our objective was to assess the relationship between adipocyte size, a common measure of cetacean adiposity, and blubber thickness in beluga whales (*Delphinapterus leucas*). We used mixed-effect generalized linear models to test how sex and blubber layer influenced this relationship. We found a significant positive relationship between adipocyte size and blubber thickness in male but not female beluga whales, suggesting sex-specific differences in fat storage or mobilization. Blubber thickness may be maintained in female beluga whales during periods with low energy reserves, for example during gestation and lactation, to preserve buoyancy, insulation, and hydrodynamism, which may be especially important when supporting swimming calves. Continuing to develop methods to assess beluga whale health will further our understanding of the impact of current and future stressors on beluga whale populations.

## Simple Summary

Beluga whales store energy from food as fat in their blubber, making blubber thickness a common measure for beluga whale health. However, blubber also functions as insulation, and helps with swimming efficiency, meaning blubber thickness may not always be reflective of energy stores. To further assess blubber thickness as a health indicator in beluga whales, we tested whether a relationship existed between blubber thickness and blubber fat cell size, which is a more direct measurement of energy storage. We examined beluga whale fat cells from different blubber depths in both sexes to determine their influence on the relationship between blubber thickness and fat cell size. We found that male beluga whales with thicker blubber had larger fat cells, indicating that blubber thickness and fat cell size can be used interchangeably to assess male beluga whale health. However, this relationship was not observed for females. Female beluga whales may require stable blubber structure even when using energy reserves, such as during pregnancy and nursing, to maintain insulation and buoyancy when supporting swimming calves. Continuing to develop methods to measure beluga whale health will be important for understanding the impact of current and future environmental changes on beluga whale populations.

## 1. Introduction

Beluga whales (*Delphinapterus leucas*) hold important roles as predators in Arctic marine ecosystems and are culturally and economically important to Inuit communities in Nunavut, Canada, that harvest them annually. However, there is growing concern over the impacts of warming temperatures and reductions in sea ice extent and duration on beluga whale populations [1,2,3]. Longer open water seasons expose beluga whales to higher levels of industrial vessel traffic and killer whale (*Orcinus orca*) predation, and warmer temperatures may alter Arctic food webs [1,4,5,6,7]. These stressors threaten the future health of beluga whale populations and the communities that rely on their harvest. Quantifying the impacts of stressors on beluga whale populations is therefore important for making future management and conservation decisions.

Body condition, the relative amount of energy reserves in an individual [8], is representative of overall health in marine mammals and can indicate the influence of stressors on individuals and be extrapolated to populations as a whole [9,10,11]. Animals in poorer body condition could indicate declines in prey abundance or shifts in preferred prey species availability, infection, or injury [1,12,13]. Body condition can also be used as a proxy for physiological stress in marine mammals. Blubber cortisol, a glucocorticoid hormone produced in response to physiological stressors [14], diffuses from the bloodstream and accumulates in adipose tissue (blubber) over time and is thought to be useful for monitoring chronic levels of stress in marine mammals [15]. For example, higher blubber cortisol concentrations were measured in narwhal (*Monodon monoceros*) during periods with higher shipping activity [16], and in threatened beluga whale populations [17]. Cortisol also plays a role in regulating the distribution and use of fat storage in marine mammals [18] and may be negatively correlated with nutritional status [19,20]. Either as an indicator of nutritional or physiological stress, changes in beluga whale body condition may impact reproductive and individual growth rates [9,10,11], and is an important parameter for understanding the effects of current and future stressors on beluga whale populations.

Energy in marine mammals is primarily stored as lipids within adipocytes in blubber tissue [21,22]. Therefore, blubber thickness has commonly been used as a proxy for body condition in both mysticetes [23,24,25] and odontocetes [26], including beluga whales [27,28,29]. For example, blubber thickness was correlated with prey availability in North Atlantic fin whales (*Balaenoptera physalus*) [24,25], North Atlantic right whales (*Eubalaena glacialis*) [23], and harbor porpoises (*Phocoena phocoena*) [26]. However, blubber has multiple structural roles in addition to energy storage, such as regulating insulation, hydrodynamism, and buoyancy [21,30,31,32,33]. Blubber thickness must therefore be maintained to support these structural roles, which can decouple the relationship between blubber thickness and energy stores [34,35,36,37,38]. For example, reductions in bowhead whale (*Balaena mysticetus*) energy stores during fasting periods were offset by increases in structural fiber density, thus preserving the streamlining and insulation function of blubber [37]. Blubber thickness in striped dolphins (*Stenella coeruleoalba*) was also unable to discriminate between non-reproductive females and females in energetically expensive reproductive stages, presumably due to the maintenance of blubber structural roles [34]. There are conflicting relationships between blubber thickness and body condition in beluga whales. Blubber thickness was not found to be strongly correlated with body condition in larger (>2.9 m) whales from the St. Lawrence Estuary, although dorsal and ventral blubber thickness had a strong correlation with body condition in smaller whales (<2.9 m) [39]. Additionally, years in which Eastern Beaufort Sea (EBS) beluga whales had reduced blubber thickness coincided with declines in preferred prey, highlighting the potential for blubber thickness to reflect environmental stressors [26,29]. Blubber is also stratified, where the inner blubber layer in marine mammals is thought to be the most metabolically active [40], the middle layer contains the majority of energy stores [41,42,43], and the outer layer is the most stable and plays the largest role in insulation [40]. Thus, the relationship between blubber thickness and energy stores may change with blubber depth. Given the stratification and multi-faceted role of blubber [21,43], and potential for sex-specific energy storage strategies [23,25,44], more work is required to understand how blubber thickness relates to body condition in beluga whales.

Other measures of blubber energy reserves, such as blubber lipid content and adipocyte metrics, have also been used to describe body condition in marine mammals [33] where body condition and nutritional status is positively correlated with lipid percentage and adipocyte size [21,33,42,45]. Adipocyte size was evaluated as one of the more effective methods for estimating cetacean body condition [33]. Compared to lipid content, adipocyte size is suggested to be the more reliable analysis method as lipids can be lost from thawing samples and extraction can vary based on the procedure being used [46]. The number of adipocyte cells is set early on during mammalian development, and subsequent changes in body condition are expected to reflect changes in adipocyte size rather than the number of cells [47]. As adipocytes change in size, blubber thickness, in theory, will increase or decrease accordingly, although adipocyte content may vary independently of blubber thickness due to the structural demands of blubber [37,42]. Despite the use of blubber thickness as a common indicator of body condition in beluga whales [27,28,29], the relationship between blubber thickness and adipocyte size as a proxy of cetacean energy stores [45] has not been formally tested in beluga whales.

Our objective was to assess the relationship between adipocyte size and blubber thickness in beluga whales. We also tested for sex- and blubber layer-specific influences on this relationship. Given that adipocyte size increases with fat deposition, we hypothesized that (1) adipocyte size would be positively correlated with blubber thickness across blubber layers and that (2) this relationship would be stronger in males than in females due to sex-specific pressures on blubber function. Results of this work provide insight into the relationship between different beluga whale blubber measures and add to our knowledge of potential beluga whale body condition indicators.

## 2. Materials and Methods

We compared adipocyte size and blubber thickness of beluga whales harvested by Inuit from Sanikiliuaq, NU, around the Belcher Islands and along the eastern Hudson Bay coast between May and July in 2021 to 2023. As part of an ongoing collaborative community-based monitoring program, blubber samples collected by hunters were provided to Fisheries and Oceans Canada for analysis. Hunters collected full-depth blubber samples below (ventral) the dorsal ridge and behind (caudal to) the pectoral fins. Blubber thickness was measured in situ from these samples. Genetic sex was determined by extracting DNA from skin tissue (DNeasy Kit, Qiagen, Hilden, Germany) and was analyzed following PCR procedures described in [48]. Blubber samples used for adipocyte measurements were separated into outer (skin-adjacent) and inner (muscle-adjacent) sections (see [41]). Samples were 0.4 × 1.5 × 2 cm in size and fixed in 50 mL of 10% neutral buffered formalin at room temperature for 48 h before being shipped in 50 mL of 70% ethanol to The Centre for Phenogenomics (Toronto, ON, Canada) where samples were processed, embedded, cut, stained, scanned, and analyzed. The area of blubber analyzed was 887,340 or 1,052,869 µm^2^ covering an average of 201 and 235 adipocytes in the inner and outer layers respectively. Samples were stained using an H&E stain (Harris hematoxylin and eosin Y) and analyzed using the Vacuole Qualification module v3.2.2 algorithm (HALO 4.2 software, Indica Labs, Corrales, NM, USA). The area of each cell within the analysis area was measured and the average adipocyte area was calculated for each sample. We further re-examined each slide for cells that were not outlined and measured correctly and re-measured the applicable cells using ImageJ (version 1.54p). Two whales had two inner blubber layer samples analyzed for mean adipocyte area, of which we took the average.

We examined the relationship between blubber thickness and adipocyte area (i.e., adipocyte area ~ blubber thickness), and the influence of sex and sampling layer on this relationship, by comparing a series of generalized linear mixed models (GLMM) in R version 2025.09.1 [49] using the “glmmTMB” package [50]. Models featured whale ID as a random effect to account for non-independence in adipocyte area measurements from inner and outer layers. We considered models featuring blubber thickness and all combinations of the additive and interactive effects of sex and sampling layer with blubber thickness. We also included a model featuring the random effect only (Null model) for comparison. We determined the best fitting models using Akaike’s Information Criterion adjusted for small sample sizes (AIC_c_) [51,52] where the top models had the lowest AIC_c_ score [52] or were within ∆ AIC_c_ 2 of the top model. We conducted model averaging if there were multiple top models using “model.avg” in the MuMIn package [53] using Akaike weights derived from the AIC_c_ values of the top models, and interpreted the coefficients of the conditional averaged model. The effects of significant interaction terms in the top models were further explored through applicable sex- or layer-specific models separately. Adipocyte area measurements in the full dataset and female-specific data were not normally distributed (Shapiro–Wilk test: *p* < 0.001) and therefore fitted using a Gamma distribution with a log link [54]. Adipocyte area measurements for male specific-data were normally distributed (*p* = 0.97) and therefore fitted using a Gaussian distribution with an identity link [54]. All models were fitted using maximum likelihood estimation [54]. We examined the top model’s residual distribution and homogeneity of model variance using the DHARMa package [55].

## 3. Results

**Figure 1:**
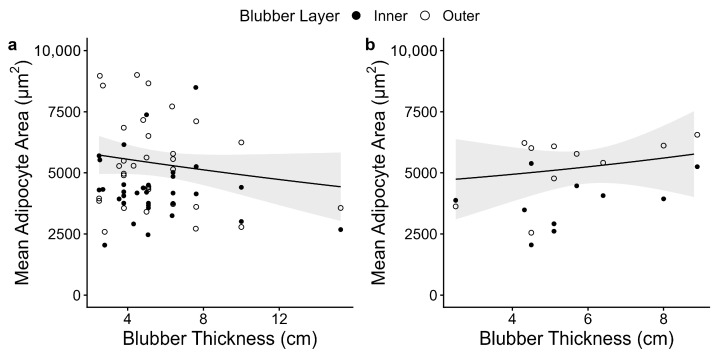
Model-predicted relationship between blubber thickness and mean blubber adipocyte area in (**a**) female and (**b**) male beluga whales. Gray band represents 95% confidence interval around values predicted from sex-specific models for predicting adipocyte area (Table 2). Points represent raw data from the inner (closed circles) and outer (open circles) blubber layers.

**Figure 2:**
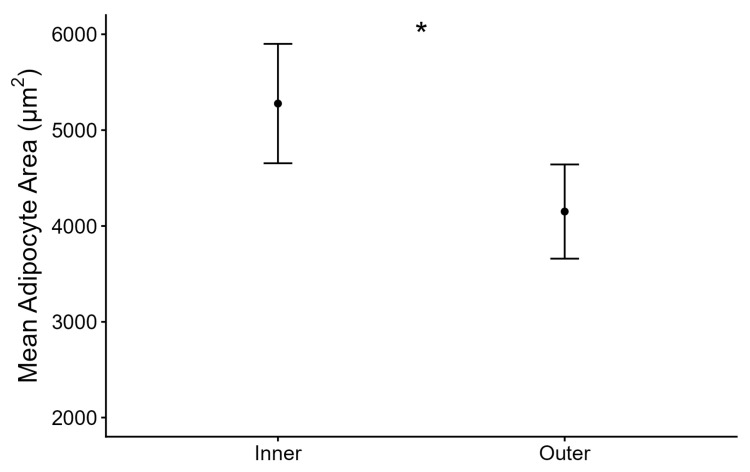
Model-predicted mean blubber adipocyte area between blubber sampling layers. Error bars represent 95% confidence intervals around predicted values. * Indicates a significant difference (*p* < 0.05).

We compared blubber thickness and adipocyte area from blubber samples collected from 42 harvested beluga whales including 32 females and 10 males. The top models explaining the relationship between adipocyte area and blubber thickness featured the interaction between blubber thickness and sex, and the additive effect of sampling layer (Table 1). The relationship between adipocyte area and blubber thickness was significantly different between female and male whales (*p* = 0.03; Table 2). We further explored sex-specific relationships between adipocyte area and blubber thickness in separate models (Table 2, middle and bottom sections for females and males, respectively). There was a significant positive relationship between adipocyte area and blubber thickness for male beluga whales (marginal R^2^ = 0.48, *p* = 0.02; Figure 1b), but no significant relationship for females (marginal R^2^ = 0.15, *p* = 0.10; Figure 1a). Given the small sample size for males (*n* = 10), we evaluated model robustness using non-parametric bootstrapping (5000 iterations) and tested for the influence of individual whales using leave-one-out sensitivity analyses. These analyses confirmed that sex-specific effects were robust and consistent in direction, with 95% confidence intervals excluding zero, despite the smaller male cohort (Table 2). Mean adipocyte area was significantly larger in the inner blubber layer compared to the outer layer (*p* = 0.001; Figure 2).

## 4. Discussion

The significant positive correlation between adipocyte area and blubber thickness in male beluga whales is consistent with our prediction and suggests adipocyte size and blubber thickness can be used interchangeably in male beluga whale health assessments. Adipocyte size has not directly been related to nutritional status in beluga whales, although adipocyte size was used to highlight a lack of primary feeding season in Hudson Bay beluga whales [41]. Furthermore, adipocyte size was used to differentiate between fasting (smaller adipocytes) and recently foraging (larger adipocytes) humpback whales (*Megaptera novaeangliae*) [45], and found to be smaller in emaciated bottlenose dolphins (*Tursiops truncatus*) [42] and harbor porpoises [21] compared to healthy adults, promoting adipocyte size as an effective indicator of body condition in cetaceans [33]. Given its multi-faceted role, the use of blubber thickness to assess cetacean energy reserves has limitations [21,34]. However, blubber thickness has been shown to respond to prey availability in other cetaceans, with male North Atlantic right whales and North Atlantic fin whales exhibiting thinner blubber during years of low prey availability [23,25]. Additionally, both male and female EBS beluga whales had thinner blubber during a year of low availability of their preferred prey, Arctic cod (*Boreogadus saida*) [29]. While based on a limited sample size (*n* = 10), our results suggest blubber thickness may be used as a proxy for adiposity in male beluga whales. More work may be required to confirm the relationship between adipocyte size and nutritional status, potentially through adipocyte size comparisons in healthy males and males in poor nutritional condition.

In contrast to males, there was no relationship between adipocyte size and blubber thickness in female beluga whales. There is no apparent primary season for fat accumulation in Hudson Bay beluga whales [41], meaning whales may have been continually feeding through spring months when our samples were collected. Blubber thickness and adipocyte sizes were also similar between male and female beluga whales. Therefore, it is unlikely for the contrasting results between sexes to be attributable to differences in energy intake or environmental stressors. Instead, our results suggest adipocyte size may vary independently of blubber thickness in females due to sex-specific factors in blubber fat storage strategies, potentially related to reproduction. In addition to energy storage, blubber regulates buoyancy, insulation, and hydrodynamism in cetaceans, which may be especially important for female beluga whales when supporting swimming calves [30,56,57].

Female beluga whales invest heavily in gestation and lactation for up to 3 years [58] and may mobilize lipids within adipocytes to provide sufficient energy during these periods, leading to a reduction in adipocyte size. For example, adipocyte size in the inner blubber layer of bottlenose dolphin mothers was found to decrease as their calves grew larger and required more resources, and was smallest in females during energetically demanding lactating periods [59]. Despite reductions in adipocyte size, blubber thickness may be maintained by collagen and elastin structural blubber fibers [37,42], which may act to preserve the function of blubber during energy-depleted periods. This is also thought to be the case for striped dolphins and bottlenose dolphins, where females in various reproductive stages (i.e., pregnant or lactating) had similar blubber thickness to non-pregnant females and males [34,60]. Thus, blubber thickness may be conserved in females despite potential decreases in adipocyte size associated with reproductive energy requirements.

It is also possible that, regardless of sex, a baseline adipocyte size is maintained throughout the blubber layer, which preserves blubber thickness [61]. In which case, catabolism of lipids and proteins from internal tissues may be used as alternative energy sources during energetically expensive reproductive periods if energy stores cannot be sufficiently replenished [62,63]. The reduction in girth and consequent reduction in body surface associated with these processes may coincide with a relative increase in blubber thickness [64]. Thus, during energetically expensive reproductive stages, female blubber thickness may be preserved, resulting in the independence of adipocyte size and blubber thickness. This is speculative, as the reproductive status (e.g., lactating or pregnant) of female beluga whales was not assessed in this study. Pregnancy rates in females were previously reported to be between 30 and 40% in beluga whale harvests from the Chukchi Sea, Alaska, and eastern Hudson Bay [65,66]. Similar rates could be expected to influence the relationship between adipocyte size and blubber thickness we observed. Incorporating reproductive status into analyses between adipocyte size and blubber thickness would be beneficial for investigating female beluga whale body condition metrics in the future.

Patterns of adipocyte size across blubber layers varies across odontocetes. Similar adipocyte patterns to our study (i.e., inner adipocytes > outer adipocytes) were observed in a healthy, recently feeding false killer whale (*Pseudorca crassidens*) [67]. However, our observations contrast with Hudson Bay beluga whales sampled seven years prior to our study [41] and bottlenose dolphins [68], where inner and outer layer adipocytes were similar in size. Given that the inner blubber layer is understood to be the most metabolically active layer and reflects diet from recent weeks to months [21,40,42,59,69,70,71,72], this difference could mean beluga whales in our study were collected during relatively productive feeding years. This productivity may be reflected in the correlation between adipocyte size and blubber thickness in male beluga whales where increased lipid accumulation resulted in adipocyte hypertrophy and consequently thicker blubber. However, this relationship may not have been observed in females due to increased energy demands of some whales associated with reproductive stages.

Despite differences in adipocyte size between inner and outer layers, sampling layer did not significantly alter the relationship between adipocyte size and blubber thickness. However, the absence of samples from the middle blubber layer represents an important limitation, as this may constrain our ability to detect depth-specific patterns that contribute most strongly to lipid storage and overall body condition. The middle section of blubber is thought to contain a majority of marine mammal energy stores [42,43], and generally feature the largest adipocyte sizes [41,43,67]. Adipocyte size in the middle blubber layer, along with inner and outer layers, was, however, not correlated with blubber thickness in false killer whales [67]. This suggests the middle layer may not significantly alter the relationship between adipocyte size and blubber thickness. It is also possible the middle layer alone may be correlated with blubber thickness in male beluga whales, and the relationship between adipocyte size in the middle blubber and blubber thickness should be assessed.

## 5. Conclusions

Here we show that blubber thickness is a valid proxy for energy stores in male but not female beluga whales. Therefore, population health assessments utilizing blubber thickness should be interpreted with caution or be sex-specific. While the use of either adipocyte size or blubber thickness can be used interchangeably as an indicator of male beluga whale health, adipocyte size analysis can be time-consuming and costly, often making processing large numbers of samples impractical [33]. In contrast, blubber thickness can be collected from harvested whales through collaboration with Inuit community harvesting programs [29,73,74], and for some populations, may be a more practical method for evaluating body condition in male beluga whales. Blubber can also be sampled from live beluga whales using remote biopsy darts. Biopsy dart samples may not always span the entire blubber depth and are therefore not a reliable method for obtaining blubber thickness measurements. We found adipocyte size and blubber thickness in males were correlated regardless of sampling depth, suggesting that remote biopsy of live whales, regardless of sampling depth, may be a suitable method for estimating male beluga whale body condition through adipocyte analysis [45]. However, utilizing alternative body morphometric indices (e.g., body girth, blubber mass, or body measurements derived from aerial photogrammetry) or developing biomarkers (e.g., hormone panels or fatty acid signatures) may be necessary for female health assessments [33].

The high variability in female adipocyte area may reflect differences in fat storage strategies between sexes, as well as the importance of maintaining the structural role of blubber related to female calf rearing. Therefore, blubber thickness may not be a strong indicator of body condition in female beluga whales. Instead, male body condition indices alone, potentially adipocyte size or blubber thickness, could be used to inform on population body condition given the competing demands associated with female blubber [29]. Monitoring changes in beluga whale body condition can provide early indicators of individual and population-level health. With rapidly changing environmental conditions in the Arctic and subsequent increases in beluga whale stressors, continuing to develop methods to assess beluga whale health will further our understanding of the impact of current and future stressors on beluga whale populations.

## Figures and Tables

**Table 1:** Results of GLMM model selection assessing the relationship between beluga whale adipocyte area and blubber thickness (BT) accounting for the influence of sex (male and female) and sampling layer (inner and outer). Whale ID is included as a random effect. Models are ranked by Akaike’s Information Criterion corrected for small sample sizes (AIC_c_), ∆ AIC_c_, and AIC_c_ weight (wt).

Model	AIC_c_	∆ AIC_c_	AIC_c_ wt
BT × Sex + Layer	1466.66	0.00	0.35
BT + Layer	1467.40	0.74	0.24
BT × Sex + BT × Layer	1468.88	2.22	0.12
BT + Sex + Layer	1469.06	2.41	0.11
BT × Layer	1469.49	2.83	0.09
BT + Sex × Layer	1470.84	4.18	0.04
BT × Layer + Sex	1471.23	4.57	0.04
BT × Sex × Layer	1473.04	6.39	0.01
Null	1475.71	9.06	0.00
BT × Sex	1476.89	10.24	0.00
BT	1477.13	10.47	0.00
BT + Sex	1478.95	12.29	0.00

**Table 2:** Parameter estimates, standard error, z-value, *p*-value, boot-strapped estimate 95% confidence intervals (CI), and range of estimates for the leave-one-out (LOO) sensitivity analysis for the averaged top models (∆ AIC_c_ < 2) and sex-specific models describing the relationship between mean blubber adipocyte area and blubber thickness (BT) in beluga whales accounting for sex (male and female) and sampling layer (outer and inner). Whale ID is included as a random effect. Females and inner sampling layers were used as reference categories. Adipocyte area measurements for the top model and female-specific models were not normally distributed and modeled using a Gamma distribution. Male-specific models were fitted using a Gaussian distribution. * Indicates a statistically significant term (*p* < 0.05).

Parameter	Estimate	SE	z-Value	*p*-Value	Bootstrap 95% CI	LOO Range (Min., Max.)
Intercept	8.70	0.10	84.54	<0.001 *	8.5, 8.9	8.7, 8.8
BT	−0.02	0.02	1.26	0.21	−0.05, 0.001	−0.03, −0.001
Male	−0.56	0.23	2.36	0.02 *	−1.0, −0.2	−0.6, −0.5
Outer	−0.24	0.07	3.59	0.001 *	−0.4, −0.1	−0.3, −0.2
BT × Male	0.09	0.04	2.17	0.03 *	0.04, 0.15	0.07, 0.1
Females						
Intercept	8.72	0.10	86.96	<0.001 *	8.5, 8.9	8.6, 8.8
BT	−0.03	0.02	−1.63	0.10	−0.05, 0.01	−0.03, −0.01
Outer	−0.21	0.08	−2.73	0.01 *	−0.4, −0.04	−0.3, −0.2
Males						
Intercept	3632.0	759.4	4.8	<0.001 *	182.6, 5563.2	3328.0, 3964.0
BT	305.7	125.7	2.4	0.02 *	71.5, 766.2	242.7, 339.5
Outer	−1509.4	444.7	−3.4	<0.001 *	−2714.0, −107.3	−1991.9, −1236.5

## Data Availability

Data generated during this study are available from https://data.mendeley.com/datasets/xg42r9t638/1 (accessed on 15 January 2026).
